# Analysis of prognostic biomarker models of TXNIP/NLRP3/IL1B inflammasome pathway in patients with acute myeloid leukemia

**DOI:** 10.7150/ijms.96627

**Published:** 2024-05-27

**Authors:** Junjie Chen, Qi Hou, Tao Chang, Jiamian Zheng, Chao Yao, Junyi He, Shengting Chen, Xiuli Wu, Zhenyi Jin

**Affiliations:** 1 The First Affiliated Hospital and Institute of Hematology, School of Medicine, Jinan University, Guangzhou, China.; 2 Department of Biochemistry, School of Medicine, Jinan University, Guangzhou, China.; 3 School of Medicine, Key Laboratory for Regenerative Medicine of Ministry of Education, Jinan University, Guangzhou, China.; 4 Department of Blood Transfusion, The First Affiliated Hospital, Jinan University, Guangzhou, China.; 5 Department of Pathology, School of Medicine, Jinan University, Guangzhou, China.

**Keywords:** Acute myeloid leukemia, TXNIP, NLRP3, biomarker, prognosis

## Abstract

**Background:** Exploring potential biomarkers for predicting clinical outcomes and developing targeted therapies for acute myeloid leukemia (AML) is of utmost importance. This study aimed to investigate the expression pattern of the thioredoxin-interacting protein (TXNIP)/nucleotide-binding oligomerization domain (NOD)-like receptor protein 3 (NLRP3) pathway and its role in the prognosis of AML patients.

**Methods:** In this study, we examined the prognostic value of TXNIP/NLRP3 pathway in AML patients using microarray data from Gene Expression Omnibus (GEO) and transcriptome data from the Cancer Genome Atlas (TCGA) to develop a prognostic model and validated the results by quantitative real-time PCR (qRT-PCR) in a validation cohort of 26 AML patients and 18 healthy individuals from Jinan University (JNU) database.

**Results:** Analysis of the GSE13159 database revealed that *TXNIP*, *interleukin 1 beta* (*IL1B*) within the TXNIP/NLRP3 pathway were significantly upregulated and *caspase1* (*CASP1*) was downregulated in AML patients (*TXNIP*, *P* = 0.031; *IL1B*, *P* = 0.042; *CASP1*, *P* = 0.038). Compared to high *NLRP3* expression, AML patients with low *NLRP3* expression had a longer overall survival (OS) in the GSE12417 dataset (*P* = 0.004). Moreover, both the training and validation results indicated that lower *TXNIP*, *NLRP3*, and *IL1B* expression were associated with favorable prognosis (GSE12417, *P* = 0.009; TCGA, *P* = 0.050; JNU, *P* = 0.026). According to the receiver operating characteristic curve analysis, this model demonstrated a sensitivity of 84% for predicting three-year survival. These data might provide novel predictors for AML outcome and direction for further investigation of the possibility of using *TXNIP*/*NLRP3*/*IL1B* genes in novel targeted therapies for AML.

## Introduction

Acute myeloid leukemia (AML) is a genetically heterogeneous disease, characterized by abnormal hematopoiesis, differentiation blockade and inadequate production of blood cells 1,2. It is the most common myeloid leukemia in adults 3,4. Previous research in our laboratory have shown that the prognosis of AML is closely related to immune checkpoints 5-7. In the era of tumor immunotherapy, the impact of inflammatory pathways on the effectiveness of anti-tumor immunity and tumor progression is increasing evident 8-10. Therefore, it is essential to study the inflammatory response in the tumor microenvironment.

Dysregulation of redox-controlled gene expression may be a common event in the pathogenesis of AML 11. It has been reported that increased levels of thioredoxin-interacting protein (TXNIP) contribute to the growth of leukemic cells 12. TXNIP, as a negative regulator of the key antioxidant system thioredoxin (TRX), is sensitive to reactive oxygen species (ROS) 13. It binds to the active cysteine residue of TRX and inhibits its antioxidative function. TXNIP also participates in other signaling pathways, playing roles in immune and inflammatory responses, glucose metabolism, and lipid metabolism. It is also involved in cell proliferation, differentiation, and apoptosis. TXNIP acts as a ligand for nucleotide-binding oligomerization domain (NOD)-like receptor protein 3 (NLRP3) 14. In the presence of high levels of ROS, elevated TXNIP protein activates NLRP3 inflammasome and activates a range of downstream genes, leading to chronic inflammatory and promoting tumor occurrence and progression 15,16. The NLRP3 inflammasome is one of the most well-known inflammasomes, consisting of NLRP3, apoptosis-associated speck-like protein containing CARD (ASC) and a CARD-domain effector protein pro-caspase-1 17. In canonical or classical activation pathways, pathogen-associated molecular pattern molecules (PAMPs) or danger-associated molecular pattern molecules (DAMPs) induce NLRP3 oligomerization, recruiting the adapter ASCs and pro-caspase-1 to assemble the NLRP3 inflammasomes 18. This ultimately leads to the self-cleavage of the pro-caspase-1, forming activated caspase-1 (CASP1), which then cleaves pro- interleukin-1β (pro-IL-1β) and pro- interleukin-18 (pro-IL-18) into their form active mature forms, IL-1β and IL-18, inducing inflammation and even cell death 19. It has been reported that the NLRP3 inflammasome is overexpressed and highly activated in AML bone marrow leukemia cells, and it is correlated with poor prognosis 20. However, the role of TXNIP/NLRP3 inflammation in AML patients remains unclear. This study aims to evaluate the potential value of inflammasome molecules in AML by examining their expression and prognostic significance. Based on the analysis from Gene Expression Omnibus (GEO), the Cancer Genome Atlas (TCGA) and our Jinan University (JNU) databases, we observed that lower expression pattern of inflammasome-related molecules was associated with better clinical outcomes. These results highlight the potential of inflammasome pathway as a promising biomarker for prognosis of AML patients.

## Materials and methods

### Microarray data from GEO

We obtained microarray data from GEO database. We considered studies were eligible according to the following standards: 1 studies with peripheral blood (PB) or bone marrow (BM) samples from healthy individuals (HIs) and AML 2 studies with information about the technology and platform utilized for studies. Based on these, we downloaded two datasets (GSE13159 and GSE12417) from the repository. GSE13159, including 542 AML patients and 74 HIs, was used to analyze relative gene expression and GSE12417, including 79 AML patients, was used to perform survival analysis.

### RNA sequence data from TCGA database

Gene expression quantification data and clinical information of AML patients were collected from TCGA database. We utilized the R package “TCGAbiolinks” to download data that include a total of 167 AML patients from TCGA. Overall survival (OS) was defined as the time from diagnosis to death or last follow-up.

### PB samples information from JNU dataset

A total of 26 PB samples were collected from the newly diagnosed AML patients at the First Affiliated Hospital of JNU database from March 1, 2014, to October 1, 2023. Additionally, 18 PB samples from age matching HIs, including 10 males and 8 females, with ages ranging from 24 to 66 years, with a median age of 36 years, were included as controls. The clinical information of the patients in the validation cohort was listed in Table 1. This study was performed in accordance with the principles of the Declaration of Helsinki and was approved by the Ethics Committee of the First Affiliated Hospital of Jinan University. All participants provided written informed consent.

### Quantitative real-time PCR (qRT-PCR)

Peripheral blood mononuclear cells (PBMCs) were separated from PB samples of the AML patients by Ficoll density centrifugation (Sigma Aldrich). Then total RNA was extracted from the PBMCs using TRIzol reagent (Invitrogen, Carlsbad, CA, USA) according to the manufacturer's instructions and was reversed transcribed into complementary DNA (cDNA) using PrimeScript™ RT reagent Kit (Takara) according to the experimental instructions. The relative expression levels of *TXNIP*, *NLRP3*, *CASP1*, and *IL1B* were measured by qRT-PCR with SYBR Master Mix (TIANGEN, Beijing, China), and *B2M* was selected as an internal control. The primer sequences for qRT-PCR are shown in Table S1. The expression levels of *TXNIP*, *NLRP3*, *CASP1*, and *IL1B* are presented as 2^-ΔΔCT^*100.

### Statistical analysis

Statistical analyses were performed using Statistical Product and Service Solution (SPSS) (version 22.0, IBM, Armonk, NY, USA), GraphPad Prism (version 8.0, CA, USA) software and R (version 4.3.2). Comparison between the differences in mRNA expression levels between HIs and AML patients were analyzed by Mann Whitney U test for non-parametric values. The log-rank test conducted by R package “survminer” was used to compare differences in Kaplan-Meier curves. The restricted mean survival time (RMST) was obtained by the “survRM2” R package. The receiver operating characteristic (ROC) curve was performed by the “timeROC” R package. A two-tailed *P* value < 0.05 was statistically significant.

## Results

### Expression and clinical features of TXNIP/NPRP3 pathway related genes in AML

**Figure 1:**
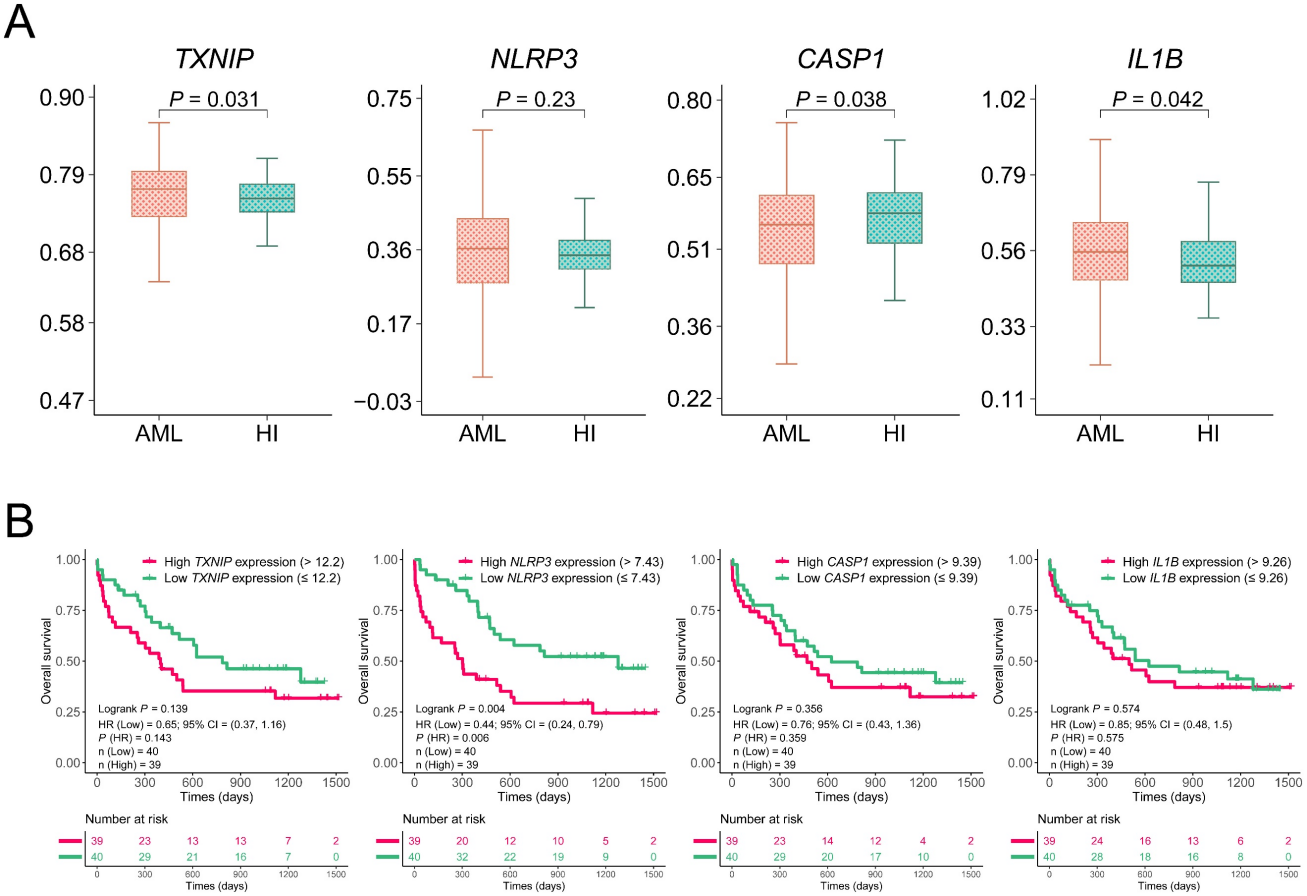
**Relative expression and OS analysis of *TXNIP*, *NLRP3*, *CASP1*, and *IL1B* in GSE13159 and GSE12417.** (A) Relative expression of *TXNIP*, *NLRP3*, *CASP1* and *IL1B* in AML patients and HIs. (B) OS analysis of high and low expression of *TXNIP*, *NLRP3*, *CASP1*, *IL1B* in GSE12417 dataset, Kaplan-Meier curves were plotted.

We initially assessed the expression levels of altered TXNIP/NLRP3 pathway in AML patients by analyzing the microarray data from the GEO database. The differentiation was analyzed between the 542 AML patients and 74 HIs from GSE13159 datasets. The expression levels of *TXNIP* and *IL1B* were significantly higher in AML patients than in HIs (*P* = 0.031 and *P* = 0.042, Figure 1A). However, the expression level of *CASP1* was lower in AML patients than in the HIs (*P* = 0.038). To further investigate the role of TXNIP/NLRP3 pathway in the clinical prognosis of AML patients, we analyzed the association between the expression levels of TXNIP/NLRP3 pathway genes and OS of microarray data from 79 AML patients in the GSE12417 dataset by Kaplan-Meier curves. Based on median of gene expression level, we divided the patients into high and low expression groups. The results demonstrated that AML patients with lower *NLRP3* expression had a longer survival time and better OS (1-year OS: *NLRP3*^low^ vs. *NLRP3*^high^ 79% vs 44%, hazard ratio (HR) = 0.44, 95% confidence interval (CI): 0.24 to 0.79, *P* = 0.004, Figure 1B). However, *TXNIP*, *CASP1*, and *IL1B* expression in the GSE12417 dataset were not statistically significant (*P* = 0.139, *P* = 0.356, and *P* = 0.574, Figure 1B).

### Lower co-expression patterns of TXNIP/NPRP3 pathway related genes are associated with favorable OS in AML patients

**Figure 2:**
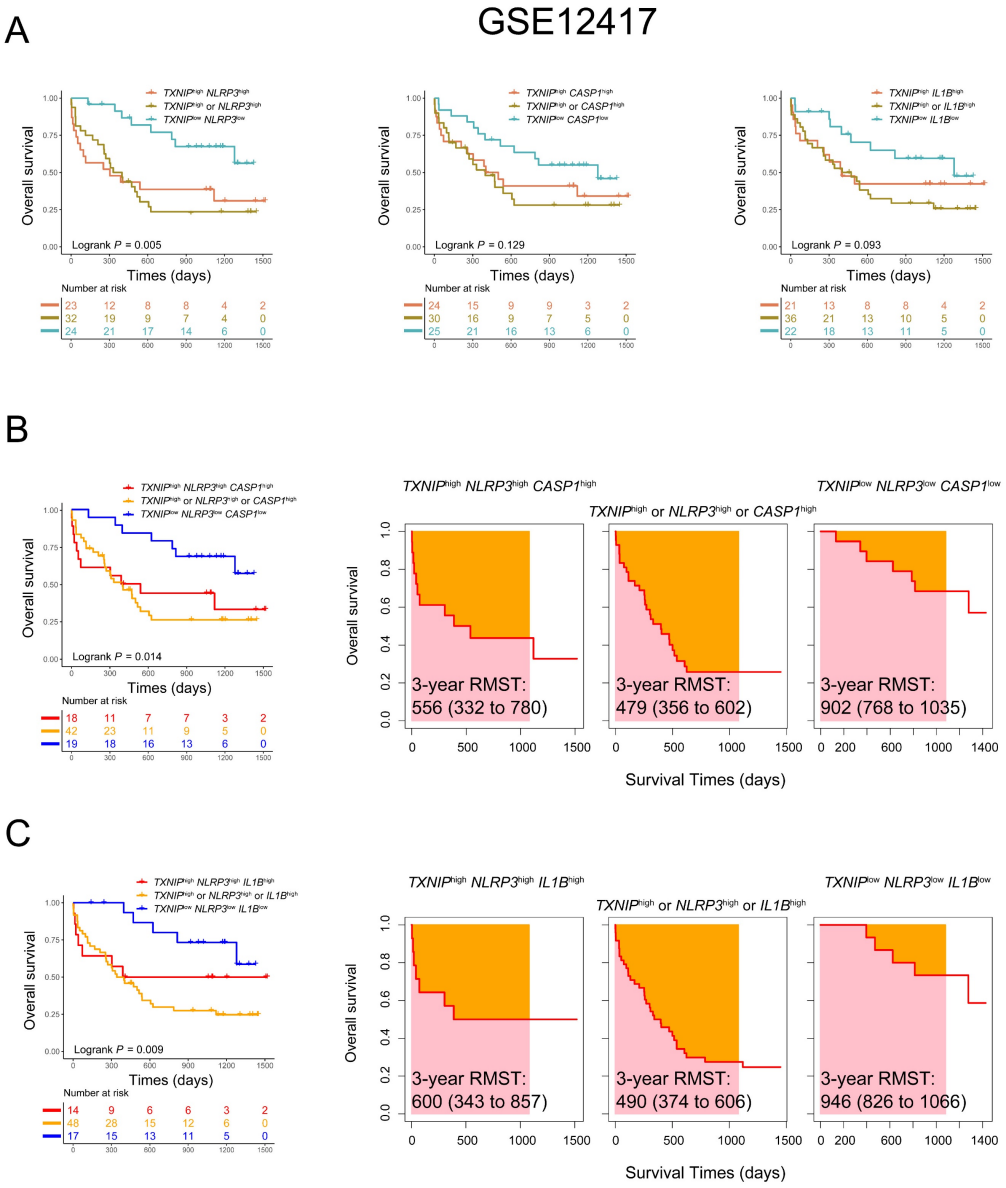
** Co-expression patterns of *TXNIP*, *NLRP3*, *CASP1*, and *IL1B* in AML patients in GSE12417 dataset.** (A) OS analysis of *TXNIP*^low^*NLRP3*^low^, *TXNIP*^low^*CASP1*^low^, *TXNIP*^low^*IL1B*^low^, and (B)*TXNIP*^low^*NLRP3*^low^*CASP1*^low^, (C) *TXNIP*^low^*NLRP3*^low^*IL1B*^low^ (left panel). The analysis of RMST, 3-year RMST was plotted (right panel).

Considering an additive effect on the outcome with multiple genes involved in the TXNIP/NLRP3 pathway, we further characterized the predictive value of co-expression patterns in AML. We found that AML patients with low expression of both *TXNIP* and *NLRP3* had better OS in comparison with those with high expression of both genes or high expression of either gene alone in GSE12417 database (1-year OS: *TXNIP*^low^*NLRP3*^low^ vs. *TXNIP*^high^*NLRP3*^high^ vs. *TXNIP*^high^ or *NLRP3*^high^, 91% vs. 48% vs. 50%, *P* = 0.005, Figure 2A). To further investigate the relationship between *TXNIP* and its related genes, we combined three genes to screen out the combination mode that best significatively predict the OS in AML patients. According to the median values of single genes, AML patients were divided into triple low, triple high, and other groups. Intriguingly, we found that patients with low expression of *TXNIP*, *NLRP3* and *CASP1* or low expression of *TXNIP*, *NLRP3* and *IL1B* had a better prognosis (1-year OS: *TXNIP*^low^*NLRP3*^low^*CASP1*^low^ vs. *TXNIP*^high^*NLRP3*^high^*CASP1*^high^ vs. *TXNIP*^high^ or *NLRP3*^high^ or *CASP1*^high^, 89% vs 56% vs 51%, *P* = 0.014; *TXNIP*^low^*NLRP3*^low^*IL1B*^low^ vs. *TXNIP*^high^*NLRP3*^high^*IL1B*^high^ vs. *TXNIP*^high^ or *NLRP3*^high^ or *IL1B*^high^, 100% vs 57% vs 50%, *P* = 0.009, Figure 2B, C). Meanwhile, RMST was used to evaluate the performance of the Kaplan-Meier curve, and we found had longer 3-year RMST in AML patients who are co-low expression of *TXNIP*, *NLRP3* and *CASP1* or *TXNIP*, *NLRP3* and *IL1B* (3-year RMST: *TXNIP*^low^*NLRP3*^low^*CASP1*^low^ vs. *TXNIP*^high^*NLRP3*^high^*CASP1*^high^ vs. *TXNIP*^high^ or *NLRP3*^high^ or *CASP1*^high^, 902 vs. 556 vs. 479 days; 3-year RMST: *TXNIP*^low^*NLRP3*^low^*IL1B*^low^ vs. *TXNIP*^high^*NLRP3*^high^*IL1B*^high^ vs. *TXNIP*^high^ or *NLRP3*^high^ or *IL1B*^high^, 946 vs. 600 vs. 490 days, Figure 2B and 2C).

**Figure 3:**
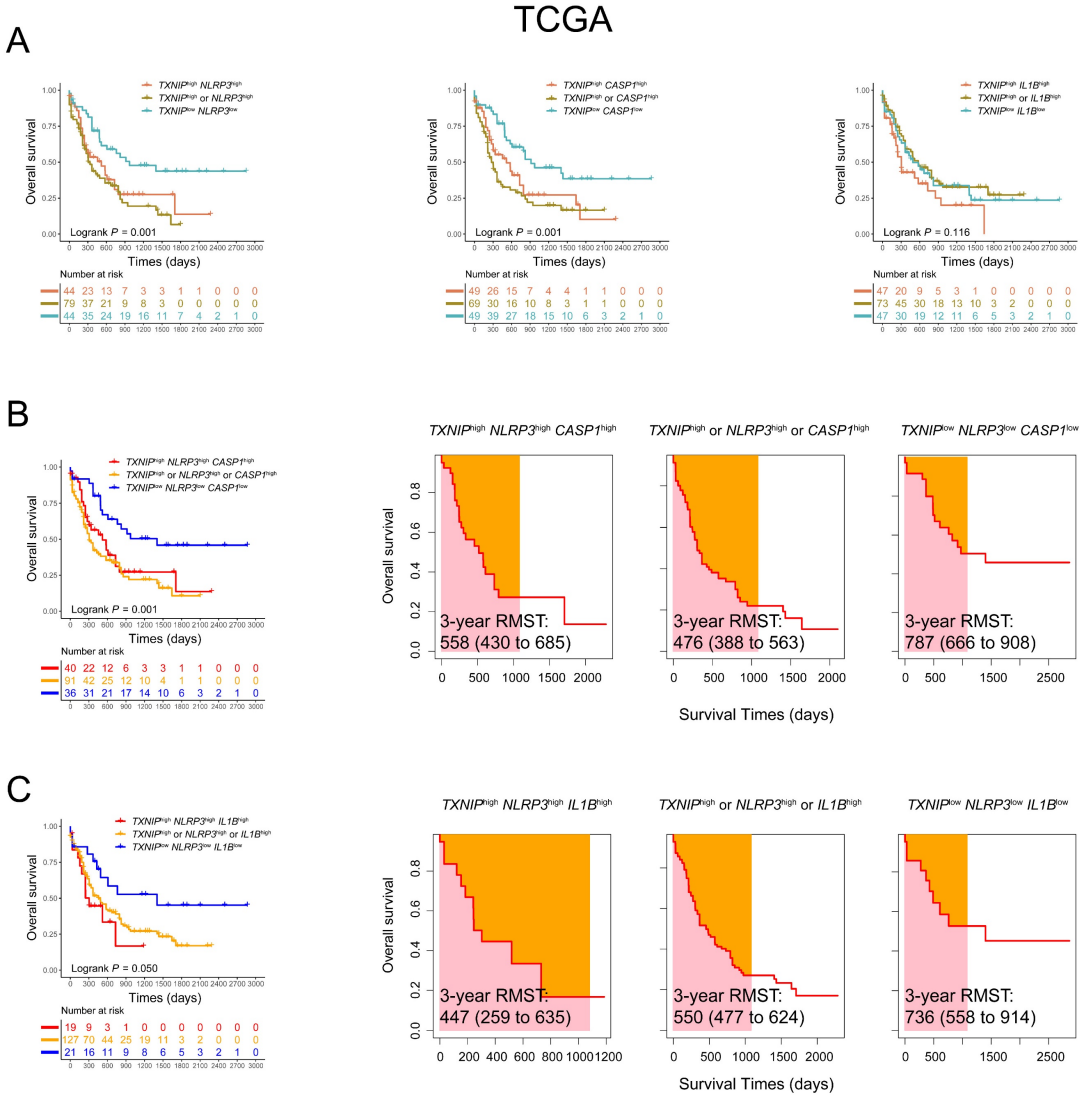
** Co-expression patterns of *TXNIP*, *NLRP3*, *CASP1*, and *IL1B* in AML patients in TCGA database.** (A) OS analysis of *TXNIP*^low^*NLRP3*^low^, *TXNIP*^low^*CASP1*^low^, *TXNIP*^low^*IL1B*^low^, and (B) *TXNIP*^low^*NLRP3*^low^*CASP1*^low^, (C) *TXNIP*^low^*NLRP3*^low^*IL1B*^low^ (left panel). The analysis of RMST, 3-year RMST was plotted (right panel).

To elucidate the prognostic importance and verify the effects of co-expression patterns of the relationship between *TXNIP* and its related genes in AML patients, we further analyzed these genes in 167 AML patients from TCGA database. Using the co-expression patterns of *TXNIP* and related genes to evaluated the OS, higher OS was observed in AML patients with low expression of both *TXNIP* and *NLRP3*, and low expression of *TXNIP* and *CASP1* compared to those with high expression of either gene or both genes (1-year OS: *TXNIP*^low^*NLRP3*^low^ vs. *TXNIP*^high^*NLRP3*^high^ vs. *TXNIP*^high^ or *NLRP3*^high^, 77% vs. 53% vs. 45%, *P* = 0.001; *TXNIP*^low^*CASP1*^low^ vs. *TXNIP*^high^*CASP1*^high^ vs. *TXNIP*^high^ or *CASP1*^high^, 82% vs. 55% vs. 37%, *P* = 0.001; Figure 3A). Furthermore, AML patients with low expression of *TXNIP*, *NLRP3* and *CASP1* and low expression of *TXNIP*, *NLRP3* and *IL1B* were significantly associated with favorable OS (1-year OS: *TXNIP*^low^*NLRP3*^low^*CASP1*^low^ vs. *TXNIP*^high^*NLRP3*^high^*CASP1*^high^ vs. *TXNIP*^high^ or *NLRP3*^high^ or *CASP1*^high^, 86% vs 56% vs 44%, *P* = 0.001; *TXNIP*^low^*NLRP3*^low^*IL1B*^low^ vs. *TXNIP*^high^*NLRP3*^high^*IL1B*^high^ vs. *TXNIP*^high^ or *NLRP3*^high^ or *IL1B*^high^, 81% vs 45% vs 54%, *P* = 0.050, Figure 3B, C). Similarly, in TCGA database, AML patients with co-low expression of *TXNIP*, *NLRP3* and *CASP1* or *TXNIP*, *NLRP3* and *IL1B* had longer RMST (3-year RMST: *TXNIP*^low^*NLRP3*^low^*CASP1*^low^ vs. *TXNIP*^high^*NLRP3*^high^*CASP1*^high^ vs. *TXNIP*^high^ or *NLRP3*^high^ or *CASP1*^high^, 787 vs. 558 vs. 476 days; 3-year RMST: *TXNIP*^low^*NLRP3*^low^*IL1B*^low^ vs. *TXNIP*^high^*NLRP3*^high^*IL1B*^high^ vs. *TXNIP*^high^ or *NLRP3*^high^ or *IL1B*^high^, 736 vs. 447 vs. 550 days, Figure 3B and 3C). These results were also confirmed in the validation cohort. Compared with patients with low co-expression of *TXNIP*, *NLRP3* and *IL1B,* those with high expression are at higher risk of death.

### Validation of the prognosis value of lower co-expression of TXNIP/NLRP3/IL1B genes in JNU dataset

**Figure 4:**
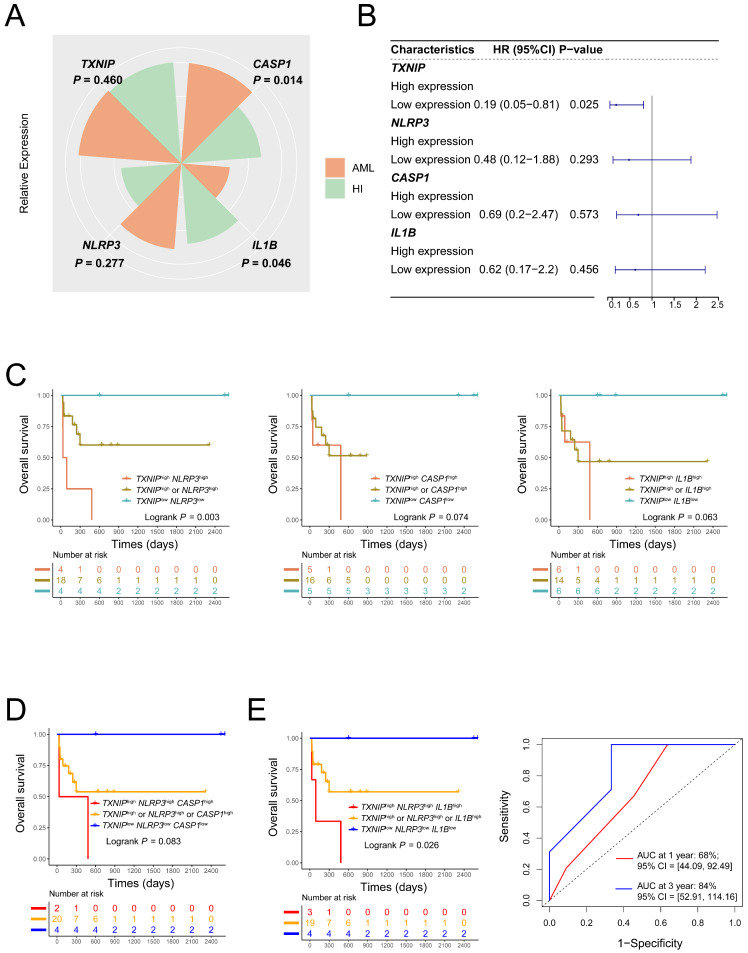
** Validation results of JNU dataset** (A) Relative expression of *TXNIP*, *NLRP3*, *CASP1* and *IL1B* in AML patients and HIs. (B) Single expression patterns of *TXNIP*, *NLRP3*, *CASP1*, and *IL1B* were plotted to be forest plot. (C) Low co-expression *TXNIP*/*NLRP3* AML patients had a better OS. (D) and (E) triple expression patterns of *TXNIP*, *NLRP3*, *CASP1*, *IL1B* and AUC of *TXNIP*/*NLRP3*/*IL1B* (right panel).

To further validate the prognostic value of TXNIP/NLRP3 pathway genes AML patients, we collected 26 AML patients and 18 HIs in our center. Compared to HIs, *CASP1* expression was significantly higher in AML patients (*P* = 0.014, Figure, 4A). In addition, the low expression of *TXNIP* had better OS in AML patients (1-year OS: *TXNIP*^low^ vs. *TXNIP*^high^, 77% vs 45%, HR = 0.19, 95%CI: 0.05 to 0.81, *P* = 0.025, Figure 4B). The co-expression patterns in JNU dataset of *TXNIP* and related genes to evaluate the OS, higher OS was observed in AML patients with low expression of both *TXNIP* and *NLRP3*, compared to those with high expression of either gene or both genes, had a better prognosis (1-year OS: *TXNIP*^low^*NLRP3*^low^ vs. *TXNIP*^high^*NLRP3*^high^ vs. *TXNIP*^high^ or *NLRP3*^high^, 100% vs 25% vs 60%, *P* = 0.003, Figure 4C). Furthermore, AML patients with low expression of *TXNIP*, *NLRP3* and *IL1B* had a longer survival time compared to those with high expression of either gene or all three genes (1-year OS: *TXNIP*^low^*NLRP3*^low^*IL1B*^low^ vs. *TXNIP*^high^*NLRP3*^high^*IL1B*^high^ vs. *TXNIP*^high^ or *NLRP3*^high^ or *IL1B*^high^, 100% vs 33% vs 57%, *P* = 0.026, Figure 4D and 4E). In conclusion, we think AML patients with *TXNIP*^low^*NLRP3*^low^*IL1B*^low^ had a better OS. Meanwhile, the area under the receiver operating characteristic curve (AUC) showed that the sensitivity of this model for predicting three-year survival was 84% (95% CI: 52.91 to 114.16, Figure 4E).

## Discussion

Inflammatory responses play a crucial role in the development and maintenance of inflammatory tumor environment, which supports tumor growth and promotes neoplastic transformation, invasion, and metastasis 21,22. The *TXNIP*/*NLRP3* pathway has been extensively studied in the context of inflammation and its inhibition by drugs, shedding light on the relationship between inflammation and disease 23,24.

We mainly focused on the prognostic biomarkers of TXNIP/NLRP3 pathway in the AML in this study. We analyzed a total of 274 AML patients from 3 different databases (GEO, TCGA, and JNU) to assess OS and validate our findings. The results showed that low expression of *NLRP3* predicted a better prognosis in AML patients in the GEO database and low expression of *TXNIP* predicted a better prognosis in AML patients in JNU database. Moreover, the co-expression of low *TXNIP* and *NLRP3* were consistently associated with improved OS across all three databases. NLRP3 is considered an important intermediator between stressful stimuli and inflammatory responses, and its deregulation has been implicated in tumor progression. Previous studies have reported increased expression levels of NLRP3 inflammasome molecules in AML, suggesting their involvement in the disease 25, 26. Basiorka *et al.* reported activation of the NLRP3 inflammasome in hematopoietic stem and progenitor cells as a critical convergence signal in myelodysplastic syndromes (MDS), which direct activation of NLRP3 complexes and CASP1 and generation of IL-1β and pyroptotic cell death 27. These findings drive pyroptotic cell death and β-catenin activation and delineation of the role of the pyroptosis in the clinical phenotype of MDS patients which suggesting new avenues for therapeutic intervention 28. There are also various mechanisms contribute to the activation of NLRP3 inflammasome, including TXNIP, calcium flux, and ROS 29. Specifically, TXNIP has been shown to bind to NLRP3 after dissociation of TXNIP from TRX in response to oxidative stress, thereby activating NLRP3 inflammasome 30. However, the functional roles of TXNIP in carcinogenesis remain controversial.

Although the expression of *TXNIP* alone, *NLRP3* alone, or co-expression of *TXNIP* and *NLRP3* cannot accurately predict the prognosis for all patients, combining *TXNIP*, *NLRP3* and other genes may yield a more precise prognostic model. Our finding demonstrated that lower co-expression of *TXNIP*, *NLRP3*, and *IL1B* were associated with better OS of AML in all three databases. These findings could serve as valuable predictor of OS for AML. Several studies have reported a correlation between dysregulated IL-1β secretion and leukemia progression and poor prognosis 31. In addition, chronic stress has been shown to enhance infiltration and proliferation of AML cells, thereby worsening OS in AML mice models 32. These highlighted the oncogenic role of NLRP3/CASP1/IL-1β signaling in AML development, with IL-1β acting as a key mediator in disease progression 33. Therefore, targeting the combination of TXNIP, NLRP3, and IL-1β with more specific pharmacological inhibitors might be more beneficial for the treatment of AML.

## Conclusions

Taken together, our study reveals that lower co-expression of *TXNIP*, *NLRP3*, and *IL1B* is associated with a favorable prognosis in AML patients. These findings provide novel insight into evaluation and the design of combination targeted therapies for AML.

## Supplementary Material

Supplementary table.

## Figures and Tables

**Table 1:** Clinical information of patients with acute myeloid leukemia

Variables	TCGA	JNU	GSE12417	GSE13159
**Total, n**	167	26	79	542
**Gender, n (%)**				
female	78 (46.8%)	10 (38.5%)	0 (0%)	0 (0%)
male	89 (53.2%)	16 (61.5%)	0 (0%)	0 (0%)
Missing	0 (0%)	0 (0%)	79 (100%)	542 (100%)
**Age, years, median(range)**				
Mean (SD)	55.0 (16.08)	52.8 (18.61)	59.8 (14.0)	-
Median (Min, Max)	58.0 (18.0, 88.0)	57.5 (20-86)	62.0 (18.0, 85.0)	-
Missing	0 (0%)	0 (0%)	0 (0%)	542 (100%)
**FAB**				
non-M3	154 (92.2%)	19 (73.1%)	79 (100%)	0 (0%)
M3	16 (7.8%)	2 (7.7%)	0 (0%)	0 (0%)
Missing	0 (0%)	5 (19.2%)	0 (0%)	542 (100%)
**Risk, n (%)**				
Favorable	33 (19.7%)	2 (7.7%)	0 (0%)	0 (0%)
Intermediate/Normal	97 (58.1%)	10 (38.5%)	0 (0%)	0 (0%)
Poor	35 (21.0%)	14 (53.8%)	0 (0%)	0 (0%)
Missing	2 (1.2%)	0 (0%)	79 (100%)	542 (100%)

Abbreviations: TCGA, the cancer genome atlas; GSE12417, gene expression omnibus series 12417 dataset; GSE13159, gene expression omnibus series 13159 dataset; JNU, Jinan University; FAB, French-American-British classification systems. *Due to rounding, not all percentages total 100%.

## Abbreviations

AML: Acute myeloid leukemia
ASC: Apoptosis-associated speck-like protein containing CARD
AUC: Area under the receiver operating characteristic curve
CASP1: Caspase1
CI: Confidence interval
DAMPs: Danger-associated molecular pattern molecules
GEO: Gene Expression Omnibus
HR: Hazard ratio
IL1B: Interleukin 1 beta
JNU: Jinan University
NLRP3: Nucleotide-binding oligomerization domain (NOD)-like receptor protein 3
OS: Overall survival
PAMPs: Pathogen-associated molecular pattern molecules
PBMCs: Peripheral blood mononuclear cells
pro-IL-18: Pro- interleukin-18
pro-IL-1β: Pro-interleukin-1β
qRT-PCR: Quantitative real-time PCR
RMST: Restricted mean survival time
ROC: Receiver operating characteristic
ROS: Reactive oxygen species
TCGA: The Cancer Genome Atlas
TXNIP: Thioredoxin-interacting protein
MDS: Myelodysplastic syndromes

## References

[1] Döhner H, Weisdorf DJ, Bloomfield CD (2015). Acute Myeloid Leukemia. New England Journal of Medicine.

[2] Zhong M, Gao R, Zhao R, Huang Y, Chen C, Li K (2022). BET bromodomain inhibition rescues PD-1-mediated T-cell exhaustion in acute myeloid leukemia. Cell Death Dis.

[3] Shallis RM, Wang R, Davidoff A, Ma X, Zeidan AM (2019). Epidemiology of acute myeloid leukemia: Recent progress and enduring challenges. Blood Rev.

[4] Bewersdorf JP, Huntington SF, Zeidan AM (2023). Cost-Effectiveness Analyses in AML: What Have We Learned, How Should This Impact Patient Care, and What Needs to Be Done in the Future?. J Natl Compr Canc Netw.

[5] Chen C, Liang C, Wang S, Chio CL, Zhang Y, Zeng C (2020). Expression patterns of immune checkpoints in acute myeloid leukemia. J Hematol Oncol.

[6] Chen C, Wang P, Mo W, Zhang Y, Zhou W, Deng T (2019). lncRNA-CCDC26, as a novel biomarker, predicts prognosis in acute myeloid leukemia. Oncol Lett.

[7] Zhong M, Gao R, Zhao R, Huang Y, Chen C, Li K (2022). Correction to: BET bromodomain inhibition rescues PD-1-mediated T-cell exhaustion in acute myeloid leukemia. Cell Death Dis.

[8] Chen DW, Fan JM, Schrey JM, Mitchell DV, Jung SK, Hurwitz SN (2024). Inflammatory recruitment of healthy hematopoietic stem and progenitor cells in the acute myeloid leukemia niche. Leukemia.

[9] Mu M, Huang CX, Qu C, Li PL, Wu XN, Yao W (2024). Targeting Ferroptosis-Elicited Inflammation Suppresses Hepatocellular Carcinoma Metastasis and Enhances Sorafenib Efficacy. Cancer Res.

[10] He Y, Qu Y, Jin S, Zhang Y, Qin L ALDH3A1 upregulation inhibits neutrophils n2 polarization and halts oral cancer growth. Oral Dis 2024.

[11] Huang D, Zhang C, Xiao M, Li X, Chen W, Jiang Y (2023). Redox metabolism maintains the leukemogenic capacity and drug resistance of AML cells. Proc Natl Acad Sci U S A.

[12] Erkeland SJ, Palande KK, Valkhof M, Gits J, Danen-van Oorschot A, Touw IP (2009). The gene encoding thioredoxin-interacting protein (TXNIP) is a frequent virus integration site in virus-induced mouse leukemia and is overexpressed in a subset of AML patients. Leuk Res.

[13] Hwang J, Suh HW, Jeon YH, Hwang E, Nguyen LT, Yeom J (2014). The structural basis for the negative regulation of thioredoxin by thioredoxin-interacting protein. Nat Commun.

[14] Deng J, Pan T, Liu Z, McCarthy C, Vicencio JM, Cao L (2023). The role of TXNIP in cancer: a fine balance between redox, metabolic, and immunological tumor control. Br J Cancer.

[15] Liang M, Chen X, Wang L, Qin L, Wang H, Sun Z (2020). Cancer-derived exosomal TRIM59 regulates macrophage NLRP3 inflammasome activation to promote lung cancer progression. J Exp Clin Cancer Res.

[16] Jin P, Zhou Q, Xi S (2022). Low-dose arsenite causes overexpression of EGF, TGFα, and HSP90 through Trx1-TXNIP-NLRP3 axis mediated signaling pathways in the human bladder epithelial cells. Ecotoxicol Environ Saf.

[17] Akira S, Uematsu S, Takeuchi O (2006). Pathogen recognition and innate immunity. Cell.

[18] Ting JP-Y, Lovering RC, Alnemri ES, Bertin J, Boss JM, Davis BK (2008). The NLR gene family: A standard nomenclature. Immunity.

[19] Liu J, Qi X, Gu P, Wang L, Song S, Shu P (2024). Baicalin Induces Gastric Cancer Cell Pyroptosis through the NF-κB-NLRP3 Signaling Axis. J Cancer.

[20] Zhong C, Wang R, Hua M, Zhang C, Han F, Xu M (2021). NLRP3 Inflammasome Promotes the Progression of Acute Myeloid Leukemia via IL-1β Pathway. Front Immunol.

[21] Binnewies M, Roberts EW, Kersten K, Chan V, Fearon DF, Merad M (2018). Understanding the tumor immune microenvironment (TIME) for effective therapy. Nat Med.

[22] Junttila MR, de Sauvage FJ (2013). Influence of tumour micro-environment heterogeneity on therapeutic response. Nature.

[23] Jia Q, Zhu R, Tian Y, Chen B, Li R, Li L (2019). Salvia miltiorrhiza in diabetes: A review of its pharmacology, phytochemistry, and safety. Phytomedicine.

[24] Zhang W, Shi C, Yao Z, Qian S (2024). Bardoxolone methyl attenuates doxorubicin-induced cardiotoxicity by inhibiting the TXNIP-NLRP3 pathway through Nrf2 activation. Environ Toxicol.

[25] Rahman T, Nagar A, Duffy EB, Okuda K, Silverman N, Harton JA (2020). NLRP3 sensing of diverse inflammatory stimuli requires distinct structural features. Front Immunol.

[26] Jia Y, Zhang C, Hua M, Wang M, Chen P, Ma D (2017). Aberrant NLRP3 inflammasome associated with aryl hydrocarbon receptor potentially contributes to the imbalance of T-helper cells in patients with acute myeloid leukemia. Oncol Lett.

[27] Basiorka AA, McGraw KL, Eksioglu EA (2016). The NLRP3 inflammasome functions as a driver of the myelodysplastic syndrome phenotype. Blood.

[28] Sallman DA, Cluzeau T, Basiorka AA, List A (2016). Unraveling the Pathogenesis of MDS: The NLRP3 Inflammasome and Pyroptosis Drive the MDS Phenotype. Front Oncol.

[29] Yoshihara E, Masaki S, Matsuo Y, Chen Z, Tian H, Yodoi J (2014). Thioredoxin/Txnip: redoxisome, as a redox switch for the pathogenesis of diseases. Front Immunol.

[30] Zhou R, Tardivel A, Thorens B, Choi I, Tschopp J (2010). Thioredoxin-interacting protein links oxidative stress to inflammasome activation. Nat Immunol.

[31] Ratajczak MZ, Bujko K, Cymer M, Thapa A, Adamiak M, Ratajczak J (2020). The Nlrp3 inflammasome as a "rising star" in studies of normal and malignant hematopoiesis. Leukemia.

[32] Liu N, Wu Y, Wen X, Li P, Lu F, Shang H (2021). Chronic stress promotes acute myeloid leukemia progression through HMGB1/NLRP3/IL-1β signaling pathway. J Mol Med (Berl).

[33] Moossavi M, Parsamanesh N, Bahrami A, Atkin SL, Sahebkar A (2018). Role of the NLRP3 inflammasome in cancer. Mol Cancer.

