# Supplementary material for: Analysis of prognostic biomarker models of TXNIP/NLRP3/IL1B inflammasome pathway in patients with acute myeloid leukemia
Source: Int J Med Sci. 2024 May 27;21(8):1438–46. doi: 10.7150/ijms.96627 (PMC11186430; doi:10.7150/ijms.96627)
Supplement: Supplementary file 1 — Supplementary table. [file ijmsv21p1438s1.pdf]

Table S1. Primer sequences.

Gene	Forward, 5'-3'	Reverse, 5'-3'
TXNIP	CTTATACTGAGGTGGATCCCTGC	TGTATCACAACATGGGCGCT
NLRP3	CATGTGGATCTAGCCACGCT	GCTCCTTGATGAGACGCAGT
CASP1	GCTGAGGTTGACATCACAGGCA	TGCTGTCAGAGGTCTTGTGCTC
IL1B	GGACAGGATATGGAGCAACAAG	TCAACACGCAGGACAGGTA

Abbreviations: TXNIP, Thioredoxin-interacting protein; NLRP3, nucleotide-binding oligomerization domain (NOD)-like receptor protein 3; CASP1, caspase 1; IL, interleukin.
